# Bloody Infiltration of Pelvic Cellular Tissue in a Doe

**Published:** 1881-07

**Authors:** 


### VII.—BLOODY INFILTRATION OF PELVIC CELLULAR TISSUE IN A DOE.

A doe which was sent from the Park was examined post-mortem, and found to have entirely normal organs, excepting its genital apparatus. It was ascertained that the pelvic cellular tissue, and especially that around the ligaments of the uterus, and around the vagina, was uniformly infiltrated with bloody fluid. The uterus was somewhat enlarged, and in its right horn a well-preserved product of conception was contained. The latter was about eight weeks of age, and showed no apparent deviation from a normally constituted fœtus. The brain of this animal was, however, not examined.

